# Exploring the Efficacy of Cognitive Behavioral Therapy for Managing Anxiety in People with Parkinson’s Disease

**DOI:** 10.3390/neurosci6040093

**Published:** 2025-09-25

**Authors:** Khaoula Elcadi, Raymond Klevor, Nissrine Louhab, Najib Kissani, Mohamed Chraa

**Affiliations:** 1 Clinical Experimental and Environmental Neuroscience Laboratory, Faculty of Medicine and Pharmacy of Marrakech, Cadi Ayaad University, Marrakech 40000, Morocco; 2 Department of Neurology, Mohammed VI University Medical Center, Marrakesh 40000, Morocco; nislouhab@gmail.com (N.L.); najibkis@gmail.com (N.K.); medchraa@gmail.com (M.C.); 3 Neuroscience Research Laboratory, Faculty of Medicine and Pharmacy of Marrakesh, Cadi Ayaad University, Marrakech 40000, Morocco; klevorraymond@gmail.com

**Keywords:** Parkinson’s disease, CBT, anxiety, depression, psychological health

## Abstract

Patients with Parkinson’s disease frequently suffer from complicated anxiety disorders that are entwined with their attitudes and behaviors. In regard to this population, cognitive behavioral therapy (CBT) has been attracting an increasing amount of attention as a potentially effective treatment for mental health issues like anxiety. CBT helps patients manage stress and improve their psychological well being through behavioral, relaxation, and cognitive techniques. Even though there is already evidence that cognitive behavioral therapy (CBT) can dramatically reduce psychological symptoms in Parkinson’s patients, more thorough research is required to determine its exact role in comprehensive anxiety treatment and prove its long-term efficacy. The purpose of this study is to examine the body of research on the use of cognitive behavioral therapy (CBT) to treat anxiety in patients with Parkinson’s disease, looking at its limitations and challenges as well as clinical characteristics, advantages, and possible behavioral and psychological impacts.

## 1. Introduction

Parkinson’s disease (PD) is the second most common neurodegenerative disorder after Alzheimer’s disease [1,2]. It can be distinguished based on a variety of non-motor symptoms, such as anxiety, sadness, insomnia, and hallucinations, in addition to motor symptoms [3,4]. These neuropsychiatric disorders may be more incapacitating than the motor symptoms experienced by a large number of people. In patients with Parkinson’s disease, the incidence of clinically significant anxiety varies from 25% to 50% [4,5,6], whereas the prevalence of depression varies greatly, ranging from 3% to 89% [4,5,7]. One of the most prevalent and severe non-motor symptoms of Parkinson’s disease is anxiety, it drastically lowers quality of life [3,8,9], and can raise the risk of suicidal thoughts and feelings [2,10]. Pharmaceuticals are the mainstay of treatment for these mental health issues; however, the majority of current treatments do not directly address anxiety as a primary goal and may have adverse effects that make day-to-day functioning more difficult [3,8,11,12]. Alternative methods, such as cognitive behavioral therapy (CBT), either by itself or in conjunction with medication, present promising ways to reduce iatrogenic effects and alleviate anxiety symptoms in individuals with Parkinson’s disease [10]. In this literature review, we will mainly explore studies investigating the effectiveness of CBT as a potentially useful treatment for anxiety and depression in Parkinson’s disease.

## 2. Prevalence of Anxiety in Individuals with Parkinson’s Disease

Parkinson’s disease is a neurodegenerative disorder characterized by the presence of non-motor symptoms alongside motor symptoms. Neuropsychiatric symptoms and indicators are prevalent in Parkinson’s disease patients over the course of the illness. These symptoms can appear similar to, or different from, those in the general population [13]. The first studies on anxiety were conducted in 1986 by Rubin et al. [14]. This study was based on a pre-established questionnaire according to The Diagnostic and Statistical Manual of Mental Disorders, 3rd Edition, Revised (DSM III R) criteria, which showed the following results: 16 out of 210 patients from the general population had episodic anxiety; 8 patients met the criteria for panic anxiety; and, among 47 patients, only 6 patients had depression. The results show that there was a different distribution of anxiety disorders, but from a general perspective, the rate of panic and anxiety was high compared to that in the general population. The study by Vázquez et al. [15] based on The Diagnostic and Statistical Manual of Mental Disorders, 4th Edition, Text Revision (DSM FII R IV TR) criteria indicated that 31 out of 131 patients had panic attacks, and only 9 patients had anxiety disorders among 387 patients from the general population. Another well-structured study focused on psychiatric interviews to explain the prevalence of anxiety disorders in Parkinson’s patients with depression and others with multiple sclerosis. The study by Scifleur et al. [14] reported that 16 patients with Parkinson’s disease suffered from depression, as did 20 patients with multiple sclerosis. The results of this study are remarkable, since 75 patients met the criteria for generalized anxiety disorder (GAD), while only 10% of the patients with MS did. This suggests that the rate of anxiety is higher in patients with Parkinson’s disease compared to those with other diseases. To further confirm this, we looked at comparative studies between Parkinson’s patients and control patients: Menza et al. [5] conducted a study on 42 Parkinson’s patients and 21 control subjects. The results showed that 12 patients had anxiety related to Parkinson’s disease, compared to only 1 patient who exhibited anxiety. Following this, we analyzed another systematic study, that of Menza and Mark [16], which was conducted in 165 patients: 104 with Parkinson’s disease and 61 control subjects. The authors found a higher rate of anxiety and depression in the Parkinson’s patients compared to the other patients. Stein et al. [17] showed that nine Parkinsonian patients were diagnosed with different types of anxiety disorders: one patient had GAD, two patients with PD had depressive episodes, and one patient had a panic disorder and social phobia; in addition, another patient had an unspecified anxiety disorder. Despite the presence of these anxiety disorders, these patients did not meet the criteria of The Diagnostic and Statistical Manual of Mental Disorders, 3rd Edition, Revised (DSM III R). In contrast to this study, Rubin et al. [18] studied Parkinsonian patients to investigate anxiety disorders that met the criteria of The Diagnostic and Statistical Manual of Mental Disorders, 3rd Edition, Revised (DSM III R). They found that 16 Parkinsonian patients among 210 patients from the normal population were diagnosed with episodic anxiety; 8 patients met the criteria for anxiety disorders, particularly panic disorders; and only 6 patients presented with major depression. Panic disorders can appear alone or can be associated with mood disorders and personality disorders [5,15].

## 3. Pharmacological Treatment for Anxiety and Depression Affecting Individuals with Parkinson’s Disease (See Table 1)

The treatments that patients undergo to address anxiety disorders are diverse and include tricyclic antidepressants, selective serotonin reuptake inhibitors (SSRIs), benzodiazepines, and buspirone. Researchers have studied pharmacological treatments used for anxiety disorders, such as Stein et al. [17], who concluded that treatments used are primarily based on antidepressants and benzodiazepines. However, there is a study that indicates that buspirone has antiparkinsonian effects. It was determined that the dose plays a crucial role in the appearance or absence of motor symptoms related to Parkinson’s disease. When the dose is around 60 mg per day, there is no problem, but when the dose exceeds 60 mg and goes up to 100 mg per day, there is a worsening that affects the physical condition of patients and subsequently contributes to a decrease in motor function with an increase in anxiety [19]. Indeed, the choice of medications used to treat anxiety in patients with Parkinson’s disease is important. For example, selective Monoamine Oxidase Inhibitors (MAOIs) are not indicated for patients who consume levodopa during treatment due to the risk of hypertensive crises. Elderly individuals are more sensitive to anxiolytic medications due to the involvement of several factors related to metabolism or chemical reactions due to other drug treatments [19]. Another important point concerns anxiety treatments for Parkinson’s patients. When anxiety attacks occur, the first treatments employed are for fluctuations. When it is necessary to use pharmacological treatments, it is advisable to apply a small dose of anxiolytics and avoid benzodiazepines, especially in the elderly. It is possible to use relaxation and therapeutic treatments, such as cognitive behavioral therapies, to reshape negative ideas and inadequate behaviors into more positive ones [20]. There is insufficient evidence for tricyclic antidepressants to reach any conclusion on the efficacy of amitriptyline for the treatment of depression in PD (see Table 1). In an open-label randomized trial, similar significant benefits were reported for amitriptyline and sertraline; the trial did not include a placebo. However, a recent review investigated the impact of antidepressants in the treatment of major depressive disorder in adults based on data from head-to-head studies and concluded that amitriptyline was more effective than other antidepressants [21].

**Table 1:** CBT vs. pharmacological treatments in Parkinson’s disease.

Criterion	Cognitive Behavioral Therapy (CBT)	Pharmacological Treatments
**Principal objective**	Reduction in anxiety and depression	Decrease in mental health symptoms (anxiety, depression)
**Type of treatment**	Group and individual psychotherapy	Medication includes tricyclic antidepressants, monoamine oxidase inhibitors (MAOIs), dopaminergic agonists, and selective serotonin reuptake inhibitors (SSRIs)
**The start of action**	A few weeks	Depending on the medication, sometimes more quickly
**Side effects**	No negative physiological consequences	Possible symptoms include nausea, insomnia, and drowsiness

## 4. Non-Pharmacological Treatment (See Table 1)

CBT is based on a specific duration of 6 to 20 sessions. The purpose of this therapy is to alleviate patients’ symptoms and find a solution to their problems. It helps patients frame their difficulties in a positive light by highlighting the experiences and events they have encountered. This enables them to understand their emotions, reactions, and ideas, and to examine their thoughts in a more rational manner, focusing on how people perceive them in relation to their situation (see Table 1).

Aaron T. Beck is the founder of this therapy. He schematized the negative thoughts associated with depression in order to find the best ways of improving mood and developing cognitive processes in patients. The effectiveness of CBT is based not only on the role of the therapist but also on the patient’s role and participation during sessions [5].

**Figure 1:**
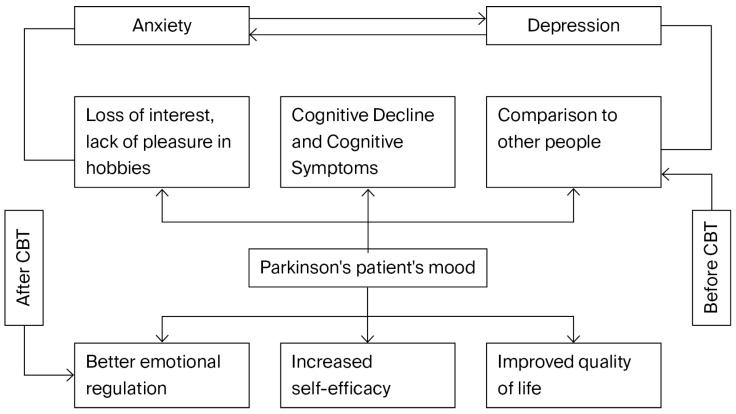
A cognitive behavioral therapy paradigm for managing anxiety and depression in Parkinson’s disease patients.

The use of CBT in psychology, from a scientific point of view, requires the psychotherapist to remain up to date with current and new approaches developed by researchers in order to adapt their practice according to the needs of patients and scientific research. CBT utilizes conversations between the therapist and the patient, based on dialogue, and requires patients to write down their thoughts as an activity. Once this has been achieved, the next step is to use techniques to analyze inappropriate thoughts and replace them with appropriate thoughts. CBT helps patients to overcome practical obstacles in life. The therapist influences patients’ daily habits and tries to build their confidence (Figure 1) and make their lives feel valuable, regardless of the stage of Parkinson’s disease. The relationship between the therapist and the patient is based on respect. CBT research suggests that patients could be more comfortable if therapists were to offer simpler, more readable, and more positive explanations. However, other CBT research suggests that the behavior of patients comes from their inner beliefs about their situation [7].

Indeed, among the CBT techniques is exposure (in the case of anxiety), which promotes acceptance, changes in attention, and full awareness. CBT is recognized for its peculiarities, which have given rise to other psychotherapies. It could be said that the most correct way to practice this therapy is to highlight the idea that beliefs that have not worked well can be removed and replaced with better developed ideas related to reality. According to Beck et al. [8], psychotherapy can be versatile [9], for example, if a patient has a combination of various mental health disorders, such as panic disorder, depression, and anxiety. This approach can help patients address each of these problems. CBT is currently classified as a psychotherapy for a wide variety of psychiatric and medical conditions according to the American Association of Psychology. It is used for anxiety disorders, depression, attention deficit/hyperactivity disorder (ADHD) in adults, chronic headaches, insomnia, irritable bowel syndrome, obsessive–compulsive disorder, panic disorder, social anxiety disorder, post-traumatic stress disorder, and bipolar disorder. Regarding depression, a randomized controlled trial (RCT) by Dobkin et al. found that the CBT group showed significant improvements in the Hamilton Depression Rating Scale (HAM-D) score, with a mean improvement of 6.53 points. In contrast, the conventional treatment group experienced a modest decline of 0.27 points (*p* < 0.0001). Over the course of a 6-month follow-up, these improvements remained. The same group’s uncontrolled pilot trial also revealed notable reductions in depression symptoms [22].

This diagram depicts a conceptual model of how cognitive behavioral therapy (CBT) improves the psychological health and mood of Parkinson’s disease patients. Patients frequently go through a cycle of emotional discomfort, marked by anxiety and sadness, prior to receiving cognitive behavioral therapy. These circumstances contribute to a negative cycle since they are interconnected and encourage one another. The patient becomes more depressed due to this vicious cycle, which is further exacerbated by issues such as cognitive deterioration, a loss of interest or enjoyment in activities, and continually comparing themself to others.

By introducing techniques that strengthen self-efficacy, improve emotional control, and improve general quality of life, cognitive behavioral therapy (CBT) breaks this pattern. Patients typically feel more capable of controlling their symptoms, develop their coping skills, and feel more involved in life after receiving cognitive behavioral therapy. These adjustments aid in mood stabilization and break the cycle of anxiety and despair. In conclusion, this graphic highlights how cognitive behavioral therapy (CBT) can improve Parkinson’s patients’ emotional experiences by addressing both the psychological mechanisms that underlie emotional pain and its symptoms.

## 5. Efficacy of CBT in Treating Anxiety Disorder in Individuals with Parkinson’s Disease

CBT is not limited to the situation that a person is experiencing, but rather to what they feel at that moment. At its heart, CBT is a way to evaluate the emotional responses of patients. In 1960, Aaron T. Beck showed that in anxious or depressed patients, automatic thoughts were the result of their impressions of themselves. However, during the 1980s, he described a pattern of automatic thinking that seemed to be specific to anxiety, with patients reporting that they sometimes did not have the ability to adapt to a situation. This led to the discovery that the way patients observe a situation or event varies from person to person, depending on their personality and the nature of their automatic thoughts [23].

It could be said that CBT helps in reorganizing thoughts. It works on emotional and cognitive aspects to correct negative thinking. It is a therapy that encourages patients to actively learn about their thoughts and develop appropriate strategies tailored to the situation.

The behavior profile of a Parkinson’s patient is generally defined by actions that are directly observable and is sometimes distinguished by mental health disorders. Cognitive, affective, and motor skills constitute the three essential components of CBT [24]. Most patients choose not to use anxiolytic drugs, despite their effectiveness in this population, and choose instead to use alternative and complementary treatments that are effective in anxiety [25]. Patients are very sensitive to the effects of drugs, and sometimes changing the dose and duration complicates their medical care.

According to a study by Borkovec and Costello [26], who examined the specific therapeutic benefits of CBT, there are significant effects on patients with GAD. There was also a meta-analysis studying the benefits of CBT in generalized anxiety disorder (GAD) [27]. In addition, Stanley et al. [28] found that there was a remarkable improvement within the CBT group: only 28% of patients who completed CBT achieved respondent status. This means that there was a 20% improvement in their behavior.

Anxiety and depression appear more frequently in patients with chronic diseases, with the percentage varying significantly [29]. The exact prevalence of these mental health disorders remains unknown [30], despite their clinical impact. Among chronic diseases associated with anxiety and depressive disorder, Parkinson’s disease stands out. One study found that 29% of patients had been diagnosed with anxiety, while 40% exhibited significant undiagnosed anxiety symptoms [5]. Additionally, 92% of the patients were diagnosed with both depression and anxiety.

## 6. Evidence Supporting the Use of Distance CBT to Treat Anxiety in Parkinson’s Disease

According to a study conducted between June 2003 and May 2004, patients were examined by the institutional committee of Baylor College of Medicine and affiliated hospitals. Patients were surveyed with regard to anxiety and depression for 27 days; the mean age of the patients was 70.8 years. The sample included 79 male and 1 female patient. The PRIMEMD questionnaire was used [31]. The questions are simple, with answers of “yes” or “no”. Three questions are about anxiety, and two questions refer to depression. Patients who had a “yes” answer were categorized into a positive group to continue the evaluation with questionnaires for each patient, including a demographic questionnaire, the Patient Health Questionnaire-9 (PHQ9), and the Beck Anxiety Inventory (BAI), in addition to the Parkinson’s Patient Health Questionnaire-9 (PHQ9) and the Parkinson’s Disease Questionnaire-39 (PDQ39), with a structured clinical interview according to The Diagnostic and Statistical Manual of Mental Disorders, Fourth Edition (DSMIV). They were assessed based on the inclusion criteria to ensure that the answers were reliable. According to Parkinson’s Patient Health Questionnaire-9 (PHQ9) and the Beck Anxiety Inventory (BAI), depression was indicated by a score of 10 or more, and anxiety by a rating of 16 or more. They also used the Mini-Mental State Exam (MMSE) to test the cognitive level of patients and other neuropsychological aspects. If participants scored 24 or more, then they could be included in the study.

The study protocol was based on CBT sessions. The patients were required to engage in one CBT session separately, followed by eight telephone sessions. The participants were evaluated directly after treatment and one month after the end of treatment. The results of this study are interesting because they show that 67.5% of the patients had symptoms of anxiety and depression, and that CBT via phone is a valid approach to detecting mental health disorders in patients. They also explain its reliability and relevance, taking a patient who had undergone 8 months of treatment for depression with a psychiatrist as an example. The patient’s issues were related to the urinary tract. The therapist proposed eight sessions of cognitive behavioral therapy (CBT) over the phone. The treatment yielded exceptional results, as it required the patient to change his ideas and perceptions regarding his problems [32]. Another study indicated that cognitive behavioral therapy (CBT) has a significant impact on reducing anxiety and depression in patients with Parkinson’s disease. The main results revealed no statistically significant effect of psychotherapy on anxiety, with a standardized mean difference (SMD) of −0.27, a 95% confidence interval (CI) ranging from −1.45 to −0.91, and a *p*-value of 0.66. However, the secondary outcome revealed that a range of psychotherapeutic therapies, including cognitive behavioral therapy and mindfulness-based techniques, were employed in this research, with significant variation observed (I^2^ = 85%) [33].

## 7. The Impact of CBT at the Functional Brain Level for Patients with Parkinson’s Disease

To confirm the importance of cognitive behavioral therapy (CBT) at the psychological level and its primary role as indicated by brain imaging, a group of researchers conducted a study in 2022 on the effect of CBT on anxiety and brain function in Parkinson’s disease patients. They studied 35 patients, selected from two groups: one that underwent cognitive behavioral therapy (CBT) and one that underwent clinical monitoring only (CMO). They used magnetic resonance imaging (MRI) as a functional tool to compare the patients before and after, and used a type of questionnaire (PAS) to evaluate anxiety. They found that patients who underwent CBT experienced changes in functional connectivity (FC) in various regions, including the temporo-frontal and parieto-occipital regions. This helped to improve the saline network (SN), the executive central network, as well as the cortico-striato-thalamo-cortical (CSTC) limbic circuits. Based on these results, it can be concluded that CBT affects anxiety by activating different areas of the brain [34].

## 8. Limitations of Cognitive Behavioral Therapy in Parkinson’s Disease Patients

Cognitive behavioral therapy (CBT) has been shown to help individuals with Parkinson’s disease feel less depressed and anxious, but its use is hampered by a number of issues. Firstly, patients’ comprehension, integration, and use of CBT procedures may be hampered by the cognitive impairments prevalent in this population, such as deficiencies in memory, attention, or executive function. Additionally, physical exhaustion and motor symptoms (bradykinesia, stiffness, and tremors) may prevent them from regularly attending programs, particularly in person. The development of a successful therapeutic partnership may also be hampered by speech and emotional expression issues, which are frequently seen in the later stages of the illness. Additionally, the patient may be less engaged in therapy and less able to carry out activities between sessions if they have related psychological symptoms such as apathy or low motivation.

## 9. Conclusions

Patients with Parkinson’s disease frequently suffer from anxiety disorders, which severely lower their quality of life. When anxiety surpasses adaptive limitations and interferes with the ability to lead a balanced life, it turns into a pathological condition, even if it can be seen as a typical reaction to motor symptoms such as trouble walking and a loss of autonomy. To lessen the intensity of non-motor symptoms, particularly anxiety, psychological interventions—in particular, cognitive behavioral treatment or CBT—should not be seen as an elective pathway but rather as an urgent necessity. CBT has proven to be a successful therapeutic approach that is easy to implement and has a positive influence on Parkinson’s patients’ behavior and brain function. Despite these encouraging results, some existing studies have small sample sizes and only short-term follow-ups. Future studies should therefore examine the long-term effectiveness and optimization of CBT procedures, employing larger and more diverse patient cohorts. Studies should also investigate customized CBT modifications that consider the cognitive and motor deficits commonly associated with Parkinson’s disease, as well as assess remote delivery strategies to increase accessibility.

## Data Availability

The original contributions presented in this study are included in the article. Further inquiries can be directed to the corresponding author.
